# The Impact of the Major Endoribonucleases RNase E and RNase III and of the sRNA StsR on Photosynthesis Gene Expression in *Rhodobacter sphaeroides* Is Growth-Phase-Dependent

**DOI:** 10.3390/ijms25169123

**Published:** 2024-08-22

**Authors:** Janek Börner, Julian Grützner, Florian Gerken, Gabriele Klug

**Affiliations:** Institute of Microbiology and Molecular Biology, Justus Liebig University Giessen, Heinrich-Buff-Ring 26-32, 35392 Giessen, Germanyflorian.gerken@mikro.bio.uni-giessen.de (F.G.)

**Keywords:** riboregulation, ribonucleases, RNase E, RNase III, stationary phase, bacterial photosynthesis, *Rhodobacter*, pigment synthesis

## Abstract

*Rhodobacter sphaeroides* is a facultative phototrophic bacterium that performs aerobic respiration when oxygen is available. Only when oxygen is present at low concentrations or absent are pigment–protein complexes formed, and anoxygenic photosynthesis generates ATP. The regulation of photosynthesis genes in response to oxygen and light has been investigated for decades, with a focus on the regulation of transcription. However, many studies have also revealed the importance of regulated mRNA processing. This study analyzes the phenotypes of wild type and mutant strains and compares global RNA-seq datasets to elucidate the impact of ribonucleases and the small non-coding RNA StsR on photosynthesis gene expression in *Rhodobacter*. Most importantly, the results demonstrate that, in particular, the role of ribonuclease E in photosynthesis gene expression is strongly dependent on growth phase.

## 1. Introduction

Most studies analyzing bacterial gene regulation look at bacteria in the exponential phase. In most natural environments, however, bacteria do not find conditions that sustain steady growth. They often have to cope with scarce nutrients, enter growth arrest, and stay in the stationary phase for long time periods. Slow-down or arrest of growth often goes along with increased resistance to abiotic stresses, like oxidative stress, temperature stress, desiccation, extreme pH, or exposure to antibiotics [1,2,3,4,5,6,7,8]. In the past, we monitored the transcriptome of *Rhodobacter sphaeroides* (recently renamed *Cereibacter sphaeroides* [9]) in different growth phases and revealed the important functions of the alternative sigma factors RpoHI and RpoHII in outgrowth after a prolonged stationary phase [10]. These alternative sigma factors are also crucial for stress resistance in *R. sphaeroides* [11,12,13,14]. Another study compared changes in the transcriptome and the proteome throughout the growth phases of *R. sphaeroides*. Surprisingly, changes in the proteome were more pronounced than changes of the transcriptome, indicating an important role of post-transcriptional processes in growth-phase-dependent gene regulation [15]. Some genes that showed growth-phase-dependent transcript levels were found to have important roles in the bacterial adaptation to stationary phase: genes for manganese uptake, polyhydroxybutyrate production, quorum sensing, and an uncharacterized alternative sigma factor (RSP_3095) [2].

More recently, we identified the small non-coding RNA (sRNA) StsR (sRNA targeting sRNA) as an important regulator of cell division in *R. sphaeroides* [16]. During stationary phase, RpoHI and RpoHII induce expression of StsR, which targets the sRNA UpsM, which is the most abundant sRNA in the exponential phase. UpsM is derived from partial transcriptional termination within the 5′ untranslated region (UTR) of the *dcw* (division and cell wall) gene cluster [17]. The binding of StsR to UpsM and to the 5′ UTR of the polycistronic *dcw* transcript leads to cleavage by RNase E and decreases the levels of *dcw* mRNAs, subsequently resulting in a retardation of cell growth. As a consequence, a strain lacking StsR reaches higher cell density in the stationary phase and resumes growth much faster in outgrowth from the stationary phase than the wild type [16].

*R. sphaeroides* is a facultative phototrophic α-proteobacterium living in aquatic environments. It performs aerobic respiration when oxygen is available and anoxygenic photosynthesis, anaerobic respiration, or fermentation in the absence of oxygen. A tightly regulated formation of photosynthetic complexes is important in avoiding photo-oxidative stress since the simultaneous presence of pigments, light, and oxygen would lead to the generation of harmful singlet oxygen. The formation of the photosynthetic apparatus is regulated by oxygen tension and light intensity, and several proteins that control the transcription of photosynthesis genes have been identified (reviewed in [18,19]). In addition, riboregulation plays an important role in photosynthesis gene expression. The action of ribonucleases and the position of structural RNA-stabilizing elements determine the stoichiometry of proteins encoded by the polycistronic *pufBALMX* operon [20]. The *puf* operon genes encode reaction center proteins (*pufL*, *pufM*), proteins of the light harvesting (LH) I complex (*pufB*, *pufA*), and an assembly factor (*pufX*). Furthermore, the sRNAs PcrZ [21], PcrX [22], asPcrL [23], and StsR [24] have been identified as regulators of photosynthesis gene expression as well as the RNA-binding proteins Hfq [25] and CcaF1 [26]. The endoribonuclease E (RNase E) has a strong effect on the levels of photosynthetic complexes and, consequently, on phototrophic growth [27]. A recent study revealed that RNase E affects the stability of mRNAs for some regulators of photosynthesis genes [28]. In stationary phase during microaerobic growth, photosynthesis genes (encoding proteins of the reaction center, light-harvesting complexes, and enzymes for the synthesis of bacteriochlorophyll and carotenoids) show a very low expression level [24] despite high amounts of photosynthetic complexes.

Since photosynthesis complexes are very stable, a new synthesis of pigments and pigment-binding proteins is only required as long as cells are dividing or changes in the environmental conditions require a switch to photosynthesis [29]. The sRNA StsR was shown to affect photosynthesis gene expression in stationary phase and to target the *rpoE* sigma factor mRNA [24]. Recently, we showed that the endoribonuclease RNase E has a strong impact on the phototrophic but not on the chemotrophic growth of *R. sphaeroides* [27,28], and also that the conserved endoribonuclease III (RNase III) affects the formation of photosynthetic complexes [30]. For these reasons, we further examined the functional roles of RNase E, RNase III, and StsR on transcript levels in exponential and stationary phase under chemotrophic (microaerobic) and phototrophic growth conditions.

Here, we show that the transcript levels of many photosynthesis genes and of genes for regulators of photosynthesis gene expression show stronger differences between microaerobic and phototrophic growth conditions in the stationary phase than in the exponential phase. The expression change of a high proportion of these genes is affected by RNase E, and some genes are affected by RNase III and/or StsR or by a combination of these RNases and StsR.

## 2. Results and Discussion

### 2.1. Effect of the Endoribonucleases RNase E and RNase III and of the sRNA StsR on Growth and Levels of Photosynthetic Complexes in Exponential and Stationary Phase

To investigate the phenotypic effects of impaired RNase E or RNase III activity or the lack of StsR, the growth of the *R. sphaeroides* wild type, the *rne^ts^* strain (substitution of the native *rne* gene with *rne-3071* from *Escherichia coli*), the *rnc**^−^*** strain (disruption of the catalytic center by amino acid exchange), and the ΔStsR mutant strain (chromosomal deletion of *stsR*) were monitored under microaerobic and phototrophic conditions.

**Figure 1:**
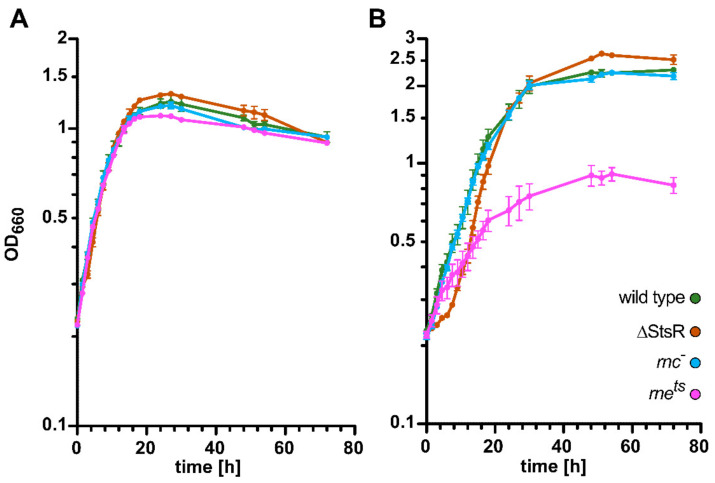
Growth behavior of the *R. sphaeroides* wild type and RNase E, RNase III, and StsR mutant strains. Bacterial strains were cultivated under microaerobic (**A**) or phototrophic (**B**) growth conditions. Optical densities were monitored at 660 nm (OD_660_) over a time span of 72 h. The mean values of independent biological triplicates of each strain are plotted. The standard deviation of the mean is indicated by the error bar.

Figure 1 demonstrates the almost-identical growth for the wild type and the mutant strains under microaerobic conditions in the exponential phase (panel A). With entry into the stationary phase, differences become visible: while the ΔStsR mutant reached a higher final optical density (OD), a lower final OD was measured for the *rne^ts^* mutant compared to the other strains. This is in agreement with our previous observation of higher ODs for the ΔStsR mutant in the stationary phase [16].

Under phototrophic conditions, a lack of RNase E is known to impede growth strongly [27,28], while the wild type and the *rnc***^−^** mutant show similar growth behavior [30] (as also seen in Figure 1B). Interestingly, the ΔStsR strain showed a prolonged lag phase, which contrasts with the three other strains. Even though ΔStsR entered exponential growth later than the other strains, it still reached a higher final optical density, as is also observed for growth under microaerobic conditions (Figure 1A). All strains besides the *rne^ts^* mutant strain have grown to higher optical densities under phototrophic conditions than under microaerobic conditions. The effect of the *rne* mutation on growth under phototrophic conditions has been described previously [27,28] and is likely due to the altered stabilities of mRNAs for important regulators of photosynthesis gene expression [28].

**Figure 2:**
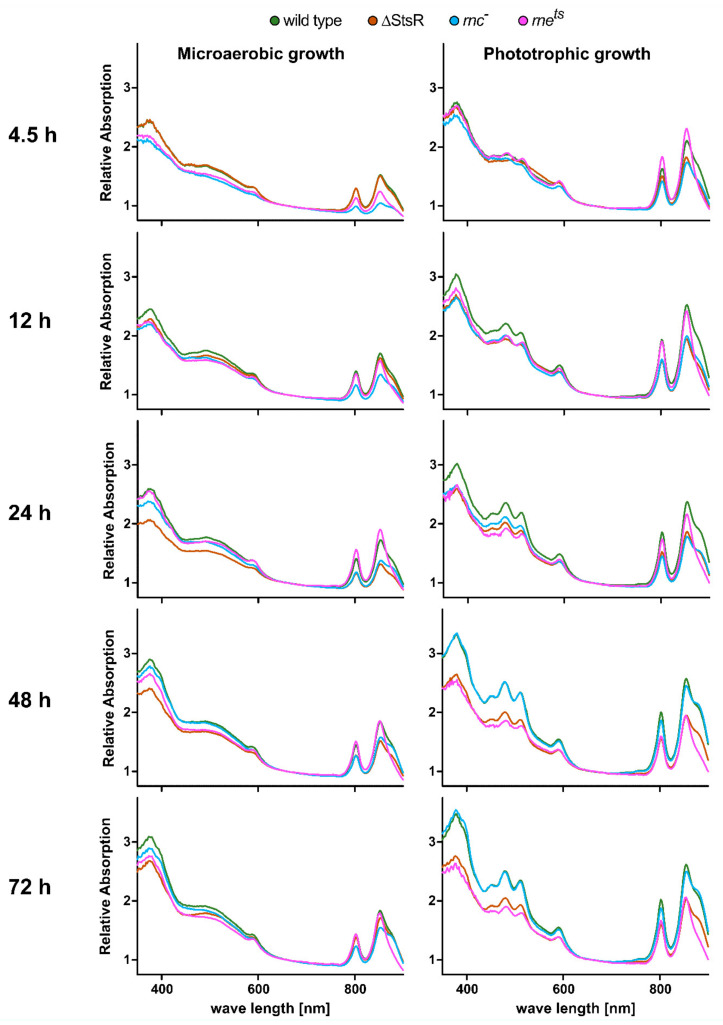
Whole-cell absorbance spectra of the *R. sphaeroides* wild type and StsR, RNase III, and RNase E mutant strains. Bacterial strains were cultivated under microaerobic (left panels) or phototrophic (right panels) growth conditions. Whole-cell absorbance spectra of independent biological triplicates were measured after 4.5 h, 12 h, 24 h, 48 h, or 72 h of growth. The mean values of independent biological triplicates (cell count normalized to OD_660_) are plotted.

To monitor the production of photosynthetic complexes, whole-cell absorbance spectra were recorded. Figure 2 shows the absorbance spectra of the wild type and mutant strains during chemotrophic or phototrophic growth in different growth phases. For chemotrophic growth in the dark, microaerobic conditions were chosen, which led to the formation of photosynthetic complexes, even in the absence of light [29].

For phototrophic growth, cultures were incubated without oxygen in the light. Bacteriochlorophylls of the most abundant LH II complex have absorbance maxima at 800 nm and 855 nm. The bacteriochlorophyll of the LH I complex absorbs at 875 nm and appears as a shoulder of the second (855 nm) LH-II-specific peak. The reaction center bacteriochlorophyll absorbs at 800 nm and 870 nm, but due to low abundance, it is not visible in the presence of the light-harvesting complexes. Carotenoids absorb in the region of 450–580 nm. Since different types of carotenoids are formed in presence or absence of oxygen, the absorbances of chemotrophic and phototrophic cultures are clearly different in this region. Other visible absorbance peaks (590 nm and 370 nm) are also caused by bacteriochlorophyll but are independent of the protein environment.

Under microaerobic conditions, the OD-normalized absorbance peaks of the wild type increased from the 4.5 h time point to the 12 h time point (in the transition from exponential to early stationary phase). At 4.5 h, both RNase mutants showed lower amounts of photosynthetic complexes, while the spectrum for the ΔStsR mutant was almost identical to that of the wild type. This can be expected since StsR shows only low expression during the exponential phase [16]. Northern blot analysis revealed that in stationary phase, StsR is more abundant during phototrophic growth than under microaerobic conditions in the wild type and the *rnc***^−^** mutant but less abundant in the *rne^ts^* mutant (Figure S1). During the later stationary phase (time points 24 h and 48 h), the ΔStsR mutant showed decreased levels of photosynthetic complexes. While the *rnc***^−^** mutant showed reduced absorbance values over all time points, the *rne^ts^* mutant showed comparable absorbance levels to the wild type after entering stationary phase (time points from 12 h–72 h).

Under phototrophic conditions, the measured absorbance values were higher than during chemotrophic growth for all strains and stayed relatively constant for the wild type during further incubation. The LH I specific absorbance at 875 nm (“the shoulder”) became more pronounced over time. In the *rnc***^−^** mutant, the LH-I- and LH-II-specific absorbances increased over time and, after 48 h, were almost identical to those of the wild type, while the absorbance levels of the ΔStsR and the *rne^ts^* mutant were clearly lower. At all time points, the LH I-specific “shoulder” was less pronounced in the *rne^ts^* mutant than in the other strains, similar to that observed for growth during microaerobic conditions.

To quantify the relative total amounts of photosynthetic complexes, we determined the bacteriochlorophyll and carotenoid levels for all strains at several time points from the exponential to the stationary phase (Figure S2).

While the measured mean values for bacteriochlorophyll show a very slight increase from time point 4.5 h to 12 h under microaerobic conditions in the wild type, the increase is much stronger and reaches maximal levels at 48 h under phototrophic conditions. A similar observation is made for the carotenoids. In agreement with our recent report for the exponential phase [30], the RNase III mutant showed reduced photopigment levels under microaerobic and phototrophic conditions at all time points tested, while the rate of increase is similar to that of the wild type (Figure S2). The photopigment concentrations measured for the RNase E mutant were close to the wild type levels under microaerobic growth conditions. In contrast, under phototrophic conditions, a very strong reduction of bacteriochlorophyll and carotenoids level is visible under phototrophic conditions as soon as the cells entered the stationary phase (data points from 12 h–72 h), and it is accompanied by the poor growth behavior of the mutant (Figure 1). Also, the ΔStsR mutant showed reduced photopigment levels, especially during phototrophic conditions. These effects became more pronounced as the cells progressed from the early to later stationary phase (12 h–48 h).

### 2.2. RNA-seq Reveals Growth-Phase-Dependent Effects of RNase E, RNase III, and StsR on the Transcriptome

**Figure 3:**
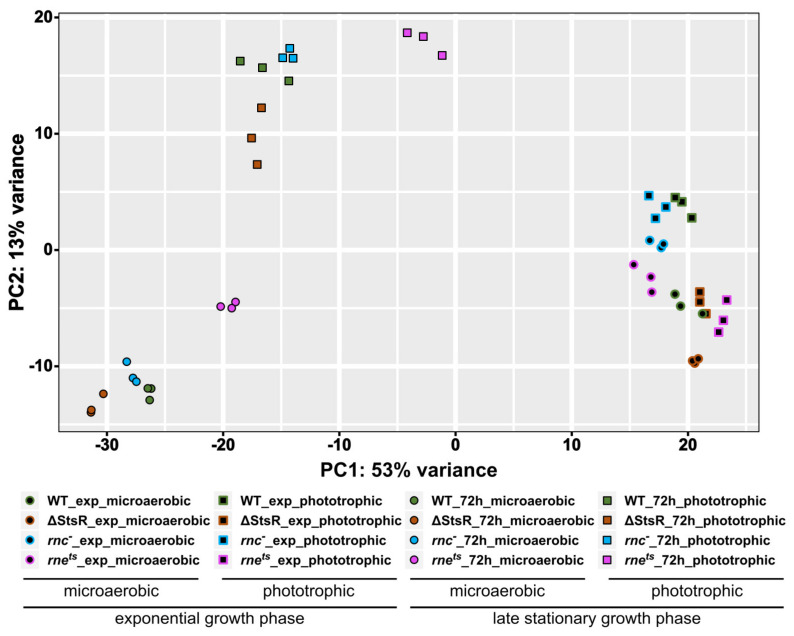
Principal component analysis of the total RNA profiling data obtained from wild type, *rne^ts^*, *rnc***^−^**, and ΔStsR mutants under various growth conditions. Total RNA of biological triplicates from each genotype was analyzed by RNA-seq. Cultures for RNA isolation and subsequent RNA-seq analysis were grown under microaerobic or phototrophic conditions to exponential or late stationary phase (72 h), as indicated by the symbol legend.

To investigate changes in the transcriptome in the wild type and mutant strains between the exponential and stationary growth phases under microaerobic or phototrophic conditions, RNA-seq analyses with total RNA were performed. The principal component analysis (PCA) shown in Figure 3 visualizes variations between the analyzed transcriptomes.

While only a minor variation within the biological triplicates was visible, a large variation between the transcriptomes from the late stationary phase and exponential phase was observed (mainly represented as difference in the *x*-axis position). Moreover, a pronounced variation between microaerobic and phototrophic transcriptomes was visible (mainly represented as difference in the *y*-axis position) when cells were in the stationary phase. In the stationary phase, the transcriptome of the *rne^ts^* mutant mapped more distantly compared to the transcriptomes of the other strains, both during microaerobic and phototrophic growth. The variation between microaerobic and phototrophic transcriptomes was much less pronounced during the exponential growth phase. Under phototrophic conditions in the exponential phase, the RNase III mutant showed the highest similarity to wild type, while the ΔStsR mutant and the RNase E mutant clustered in close proximity to each other but distantly from the wild type.

### 2.3. Growth-Phase-Dependent Effects of RNase E, RNase III, and StsR on Expression of Photosynthesis-Related Genes

**Figure 4:**
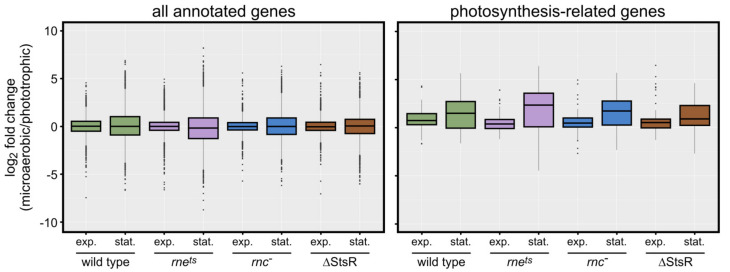
Box plots showing the distribution of the log_2_ fold changes of all annotated genes (**left panel**) and of 89 photosynthesis-related genes as outlined in the main text (**right panel**) from microaerobic to phototrophic growth conditions. The log_2_ fold changes are taken from the corresponding DESeq2 analyses as described in the Materials and Methods section.

While the influence of light and oxygen availability on the expression of photosynthesis genes has been studied intensely in recent decades, less is known about the influence of growth phases on the expression of photosynthesis genes. We first visualized the expression of 89 annotated photosynthesis-related genes in the EMBL-EBI QuickGO database, which were found using the following photosynthesis-related GO (gene ontology) terms: 0015979: photosynthesis; 0015995: chlorophyll biosynthetic process; 0016117: carotenoid biosynthetic process; 0030076: light-harvesting complex; 0030494: bacteriochlorophyll biosynthetic process; 0036070: light-independent bacteriochlorophyll biosynthetic process; 0033014: tetrapyrrole biosynthetic process; 0006782: protoporphyrinogen IX biosynthetic process; 0006783: heme biosynthetic process [31]. The box plots in Figure 4 compare the expression change (log_2_ fold) of the genes between microaerobic and phototrophic growth.

Due to the normalization by DESeq2, the average expression changes of all annotated (4450) genes was almost unaffected by the growth phase (exponential or stationary) or the strain background (left panel), resulting in a median close to 0 (left panel). However, in the stationary phase, the log_2_ fold changes showed clearly larger variations. When analyzing the photosynthesis-related genes (right panel), the median was slightly higher than 0 during exponential growth for all strains. On the other hand, the median was even higher during the stationary phase, and the variations of the log_2_ fold changes were much larger than in exponential phase and were also larger compared to all genes in the stationary phase (left panel). The highest variation for the expression of photosynthesis-related genes in the stationary phase is observed for the *rne^ts^* strain, in agreement with earlier observations [28]. In summary, the box plots demonstrate an influence of growth phases on photosynthesis-related genes.

The box plots in Figure S3 show the average log_2_ fold changes of gene expression in the stationary phase compared to the exponential phase. The general distribution of expression changes for all genes was larger during microaerobic growth compared to phototrophic growth (median values were close to 0). Looking only at the photosynthesis-related genes, the median was shifted to negative values for all strains and conditions but not for the *rne^ts^* strain during phototrophic growth (median slightly higher than 0). The spread between log_2_ fold change values was higher during microaerobic growth than during phototrophic growth; this difference was smaller in the *rne^ts^* strain than in the other strains.

**Figure 5:**
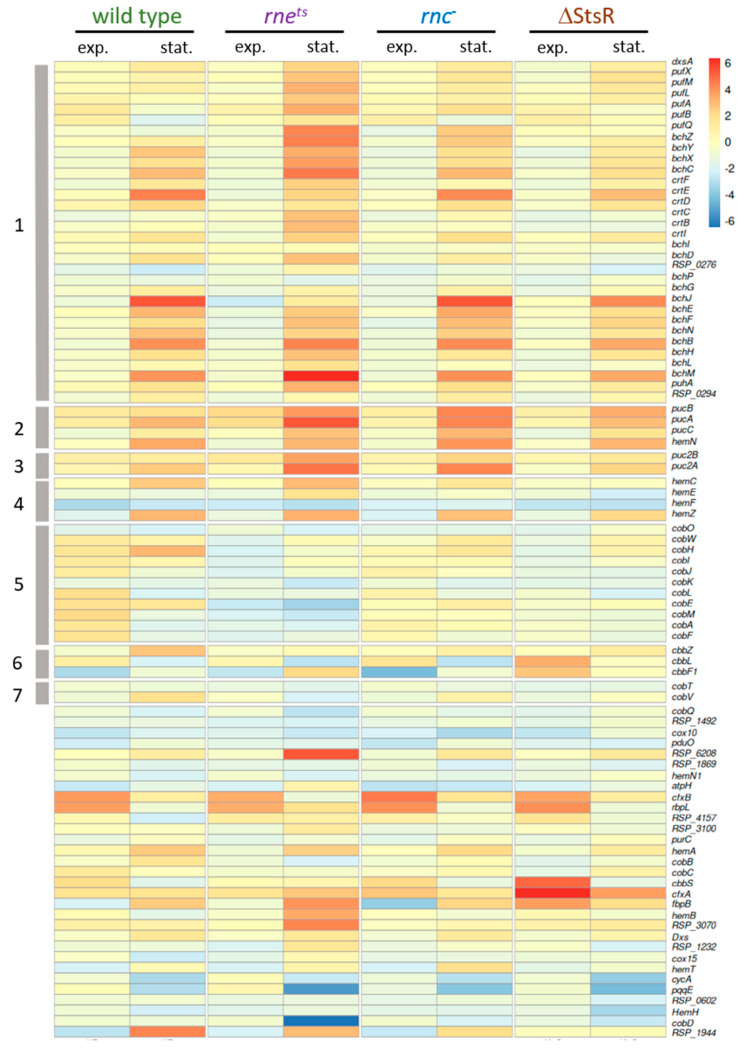
Heatmap visualizing the expression changes between microaerobic and phototrophic growth of photosynthesis-related genes (as log_2_ fold changes based on the DESeq2 analysis of the transcriptomes). As inclusion criterion for the heatmap, the plotted genes showed a significant expression (adjusted *p*-value < 0.05) in at least one comparison. Genes within one cluster (gray bars numbered 1 to 7, described in the main text) are localized adjacent to each other on the *R. sphaeroides* chromosome. The log_2_ fold change is depicted in a color code from blue (negative) to red (positive).

Furthermore, we also visualized the expression in response to growth phases and growth conditions via heatmaps for the 89 selected photosynthesis-related genes (Figure 5).

First of all, the heatmap visualizes much stronger expression changes between microaerobic and phototrophic conditions in the stationary phase compared to the exponential phase in all strains, as also revealed by the box plots. The strongest deviation from the wild type pattern is again observed for the *rne^ts^* strain. Genes that are localized together on the chromosome are marked (clusters 1–7, gray bars). While some of the genes in clusters 1, 2, and 3 are known to be co-transcribed [32,33,34], not all co-localized genes which occur in the heat map are also co-transcribed (some are not shown due to the cut-off criteria for base mean and *p*-value). Clusters 1–3 comprise the genes for the proteins of the photosynthetic complexes (*puf* and *puc* genes) and the genes for bacteriochlorophyll (*bch*) or carotenoid (*crt*) synthesis. The expression pattern of these genes during exponential growth was most similar between wild type and the ΔStsR strain, while the expression change in the ribonuclease mutants was stronger for most of these genes. In the stationary phase, the *rne^ts^* strain showed the strongest differences to the wild type; in particular, the *puf* genes showed stronger expression change.

Cluster 4 comprises 4 genes for heme biosynthesis; cluster 5 comprises 11 genes; and cluster 7 comprises 2 genes for cobalamin synthesis. Heme and cobalamin are required for bacteriochlorophyll synthesis. Cluster 6 comprises three *cbb* genes encoding proteins of the Calvin–Benson–Bassham (CBB) cycle. The expression changes of cluster 4 and 5 genes between microaerobic and phototrophic growth in the exponential phase for the wild type was less than that for the RNase mutants.

Genes that are not included in clusters 1–7 are localized at different positions of the chromosome. RSP_6208 has a role in porphyrin synthesis and shows high expression change only in the *rne^ts^* strain in the stationary phase. The genes *cfxA* and *cfxB* encode fructose-6-P aldolase and show similar and remarkably strong expression changes between the different strains, despite their localizations in different regions of the chromosome.

### 2.4. Effect of Growth Conditions and Growth Phases on Other Functional Groups of Genes

Next, we analyzed the average change in gene expression between microaerobic or phototrophic growth in the exponential and stationary phases for the other functional groups of genes. While we observed increased expression changes of the photosynthesis gene cluster (38 genes: clusters 1–3 of the heat map in Figure 5) during exponential growth in all tested strains, the expression of motility genes (63 genes, *RSP_0032-0083*) decreased in the wild type but increased in the *rne^ts^* mutant, and was only weakly affected in the *rnc***^−^** and ΔStsR strains (Table 1). A strong effect of RNase E on expression of the motility genes in *R. sphaeroides* has previously been observed [28]. Growth phases had a similar strong effect on the expression changes of motility genes, as observed for photosynthesis genes. Expression changes of the *dcw* genes (division and cell wall synthesis, 24 genes, *RSP_2095-2117*) or *rps* and *rpl* genes (for ribosomal proteins, 37 genes, *RSP_1705-1740*) showed a lower growth-phase-dependent response. In the stationary phase (72 h after inoculation), photosynthesis genes showed stronger differences between microaerobic and phototrophic incubation in all strains, as also demonstrated by the box plots (Figure 4) and the heatmap (Figure 5). The same was true for *rps* and *rpl* genes. Strong differences in expression changes between exponential and stationary phase were also observed for motility genes. Differences in their expression levels were less pronounced in the wild type in the stationary phase but were more pronounced in the ΔStsR mutant. Motility gene expression was also strongly affected by RNase E but in the opposite manner to photosynthesis gene expression. The expression changes of motility genes differed from those in the wild type in all mutant strains. These data demonstrate that the strong effects of growth conditions and growth phases are restricted to some functional groups of genes and can be very different among those groups (photosynthesis genes versus motility genes).

### 2.5. Influence of RNase E, RNase III, and StsR on the Growth-Phase-Dependent Expression of Selected Photosynthesis Genes and Their Regulators

For a more detailed analysis of the effect of growth phases on photosynthesis gene expression, we focused on the genes shown in Table 2. We selected these genes as representatives of genes with a direct role in the formation of photosynthetic complexes (group A) or because of their well-known functions in the regulation of photosynthesis genes (groups B–F).

The selected genes are grouped according to their function. Group A genes (part of the photosynthesis-related genes, representing different functions) are required to build photosynthetic complexes: *hemA*, *hemZ*, and *bch* genes are required for the synthesis of bacteriochlorophyll; *crt* genes for the synthesis of carotenoids; *puf* genes encode proteins of the reaction center and of the LH I complex; *puc* genes encode proteins of the LH II complex; RSP_0290 is required for the assembly of the LHI complex; and *fbc* genes encode the subunits of the cytochrome *bc1* complex for photosynthetic electron transport. Group B genes encode known protein regulators of photosynthesis gene expression [18]; group C genes encode RNase E, RNase III, and the exoribonuclease polynucleotide phosphorylase.

While RNases mostly destabilize their target mRNAs, cleavage can also generate more stable processing products (e.g., [35,36,37]). Target cleavage is often promoted by sRNAs and/or RNA-binding proteins like Hfq [38,39,40]. Furthermore, ribonucleases can generate 5′- or 3′-derived sRNAs with important regulatory functions [41,42,43,44]. RNase E is one of the most important ribonucleases in mRNA decay in bacteria [45,46,47]. More recently, a large impact of RNase III in bacterial gene expression in a wide range of bacteria has been reported [48,49,50,51,52,53,54], and RNase III-CLASH (UV cross-linking, ligation, and sequencing of hybrids) has been established as a potent, novel tool for uncovering important new sRNA regulatory networks [55,56].

Group D genes encode important alternative sigma factors with known roles in stress responses and/or stationary phase [2,10,11,57,58]; group E genes encode small non-coding RNAs that influence the formation of photosynthetic complexes [16,21]; and group F genes encode small RNA-binding proteins, affecting the formation of photosynthetic complexes in *R. sphaeroides* [25,26,59]. RNA-binding proteins can control the regulatory activity of sRNAs in bacteria [60,61,62,63,64].

All group A genes except for *hemZ* (encoding coproporphyrinogen III oxidase) showed increased expression during phototrophic growth compared to microaerobic growth in exponential phase. This is also true for *bch* and *crt* genes (not included in this table). Except for the *puf* genes, all these genes showed much stronger expression changes in the stationary phase. The expression level of almost all of these genes was dependent on RNase E (in some cases, only under one of the growth conditions). Note that we defined RNase-dependency as at least a two-fold difference in the expression change between the wild type and mutant strain. Some of group A genes were RNase E- and RNase III-dependent in the stationary phase. *hemA* and *crtE* were the only group A genes with StsR-dependent expression.

**Figure 6:**
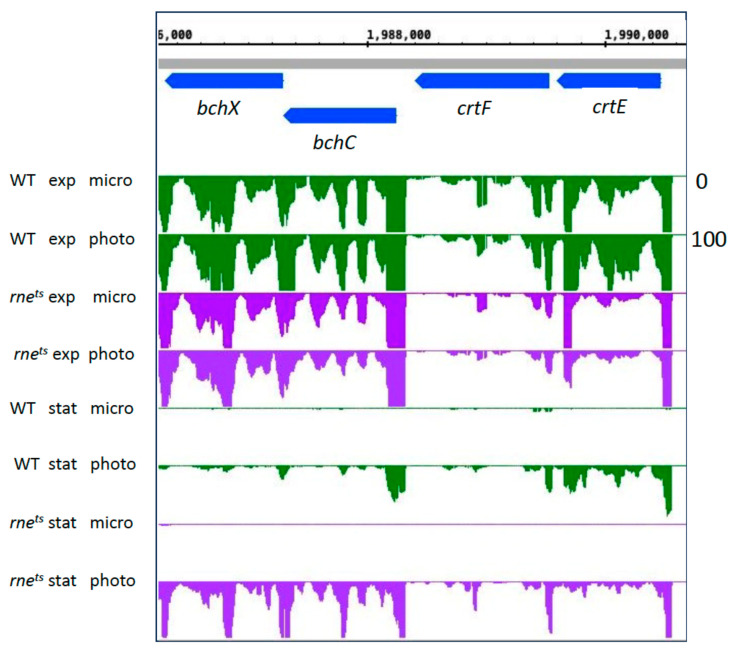
Normalized read coverage plot taken as a screenshot from the Integrated Genome Browser (IGB) showing reads for selected genes for bacteriochlorophyll (*bchCX*) and carotenoid (*crtEF*) syntheses. The *y*-axis count scale is indicated on the right side.

Figure 6 shows a read coverage plot for the expression of the *crtE*-*crtF* and *bchC*-*bchX* genes in the wild type and the *rne^ts^* mutant.

The plot demonstrates that the higher expression changes between microaerobic and phototrophic conditions in the stationary phase are due to very low expression under microaerobic conditions in the stationary phase in both the wild type and mutant strain. Stationary phase had a much stronger negative effect on the expression of the photosynthesis gene cluster during microaerobic growth than during phototrophic growth. In the stationary phase, during phototrophic growth, the expression of these *bch* and *crt* genes was clearly stronger in the RNase E mutant. This is in agreement with the expression pattern visualized in the heatmap (Figure 6). However, Figure S2 demonstrates lower bacteriochlorophyll and carotenoid levels in the *rne^ts^* mutant compared to the wild type in the stationary phase. The pigments quantified in Figure S2 are bound to proteins since free pigments are not stable. Genes for pigment-binding proteins (*puf*, *puc*) also show higher expression in the *rne^ts^* mutant compared to wild type in the stationary phase (see Supplementary Table S3). We conclude that other, as-yet-unidentified factors are responsible for lower pigment levels in the mutant in the stationary phase. As the biochemical pigment synthesis pathways are composed of several enzymes responsible for the synthesis of different intermediates, the upregulation of single pigment-synthesis-enzyme-encoding mRNAs (like some *bch* and *crt* mRNAs in the *rne^ts^* mutant) may not result in increased production of photosynthetically functional end products.

**Figure 7:**
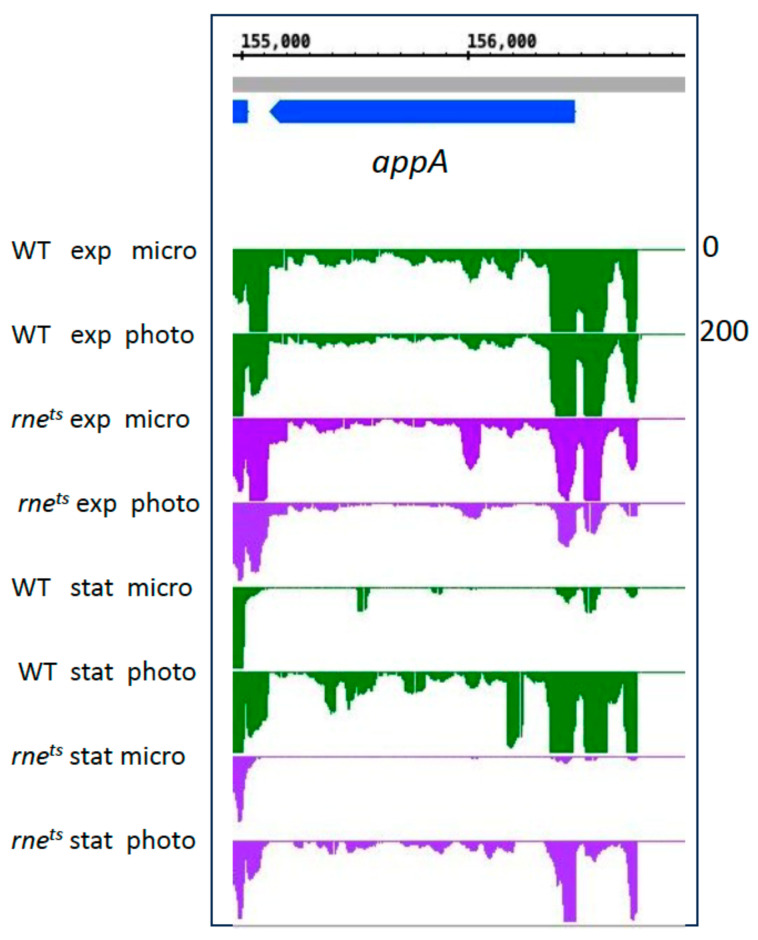
Normalized read coverage plot taken as a screenshot from the Integrated Genome Browser (IGB) showing reads for the *appA* gene, encoding an important regulator for the oxygen- and light-dependent expression of photosynthesis genes. The *y*-axis count scale is indicated on the right side.

Group B genes encode important regulators for the oxygen-dependent expression of photosynthesis genes [18,65,66,67]. AppA not only senses oxygen through a heme group but also light through its BLUF domain [68,69,70]. Most of these group B genes showed only low expression changes in the exponential phase. *fnrL* and *ppsR* expression decreased in the wild type in the exponential phase. In particular, *appA* showed a strong increase in expression in phototrophic conditions in the stationary phase, although the total expression was less than that of the wild type (Figure 7).

The expression pattern of the *appA* gene in the WT is similar to the expression of the *bch* and *crt* genes, as shown in Figure 6. In contrast to those genes, expression of *appA* was affected by RNase E during microaerobic and phototrophic growth. This effect is much more pronounced in the stationary phase. The effect of RNase E on the stability of the *appA* transcript was described previously [28]. Our data also reveal effects of RNase III and StsR on *appA* expression. For the *ppsR* gene, encoding the anti-repressor to AppA, we observed only minor expression changes and no strong influence of the strain backgrounds (Table 2).

An effect of RNase E on *prrA* expression was detected exclusively in the stationary phase (Table 2). PrrA and PrrB form a two-component system important for the redox-regulation of photosynthesis genes. FnrL, an activator of photosynthesis gene expression at low oxygen tension [18], showed lower expression during microaerobic growth independently of the growth phase. Our data hint at a major role of AppA in the growth-phase-dependent expression of photosynthesis genes.

Group C genes encode RNase E, RNase III, and the exoribonuclease polynucleotide phosphorylase. A previous study showed that the protein levels of RNase E and RNase III decrease in the late growth stages [71]. Our data indicate that both *rne* and *rnc* are less expressed under phototrophic conditions compared to microaerobic growth in the exponential phase. In the stationary phase, *rnc* expression is increased during phototrophic growth (Table 2). An effect of all three RNases on the transcriptome of *R. sphaeroides* has previously been demonstrated [27,28,30,71]. This study underlines that growth conditions affect gene expression in *R. sphaeroides* not only through transcriptional regulators like AppA but also through their effect on the expression of RNases. Furthermore, the data indicate that the RNases might regulate each other. Since the regulation of RNase genes has not been studied in *R. sphaeroides*, the underlying mechanisms are not known.

**Figure 8:**
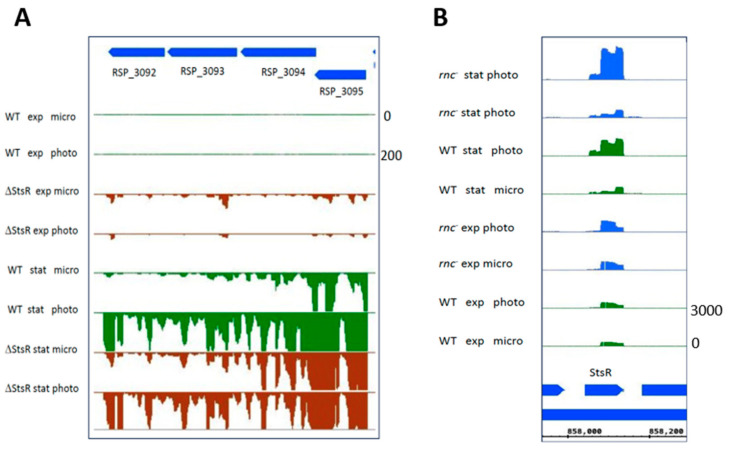
Normalized read coverage plots taken as a screenshot from the Integrated Genome Browser (IGB) showing reads for the *RSP_3092-3095* genes (**A**) and the StsR-encoding gene (**B**). The *y*-axis count scale is indicated on the right side of each panel.

Group D genes encode important alternative sigma factors with known roles in stress responses and/or stationary phase [2,10,11,57,58]. The RpoH sigma factors, especially RpoHI, play an important role in the adaptation to the stationary phase [10]. *rpoHI* showed higher expression during phototrophic growth, but only in the exponential phase. *rpoHII* levels moderately increased under phototrophic conditions only in the stationary phase and were affected by StsR (Table 2). *rpoE* showed much a stronger response to growth conditions in the stationary phase in all strains (Table 2). We demonstrated before that RpoHI, RpoHII, RpoE, and StsR are part of a regulatory network affecting photosynthesis expression [24]. As a consequence, changes in the expression of one regulatory factor will not only affect its own regulon but also the expression of genes that are affected by other regulators. Expression of the RSP_3095 sigma factor is strongly induced in the stationary phase [2]. Our data reveal a strong response of RSP_3095 to growth conditions in the stationary phase that is dependent on RNase E, RNase III, and StsR (Table 2). *RSP_3095* is most likely co-transcribed with the *RSP_3092-3094* genes of unknown function (Figure 8A). All these genes showed much stronger expression in the stationary phase than in the exponential phase. While expression in the wild type was much stronger during phototrophic growth in the stationary phase, expression in the ΔStsR mutant was similar under microaerobic and phototrophic conditions.

Interestingly, StsR (group E [24]) showed higher abundance under phototrophic conditions only in the stationary phase. This effect was even stronger in the *rnc***^−^** strain (Figure 8B). Considering the influence of StsR on the growth of *R. sphaeroides* [16], this emphasizes an important role of StsR not only in adaptation to growth phases but also in adaptation to growth conditions. Furthermore, the expression of StsR is not only controlled by RpoHI, RpoHII and RNase E, as reported previously [26], but also by RNase III. These data demonstrate again that complex networks of proteins and RNAs have major roles in the adaptation of bacteria to growth conditions and growth phases.

UpsM plays an important role in controlling the growth of *R. sphaeroides*. It is a target of StsR, and cleavage by RNase E has been demonstrated [16]. This RNase E-dependency is observed in our dataset only in the stationary phase, i.e., when UpsM is highly expressed (Table 2). UpsM expression is also influenced by RNase III, but the underlying mechanism (to date) is not known.

Alongside StsR, group E contains another sRNA with a known function in photosynthesis gene regulation: PcrZ [21]. Interestingly, PcrZ expression was strongly affected by StsR in the exponential phase, when StsR showed only low abundance. During the stationary phase, RNase E influenced PcrZ levels. Cleavage of PcrZ by RNase E has been demonstrated previously [27]. PcrZ showed higher levels in the stationary phase, especially in the strain lacking StsR [24]. Thus, PcrZ is another part of the regulatory network by which StsR acts on gene expression. A previous study also revealed an influence of stress conditions and growth phases on the generation of sRNAs that are derived from the 5′ or 3′UTRs of mRNAs by RNases [72]. Since some of these sRNAs, like UpsM, are important regulators [16], this is another pathway how RNases affect gene expression.

The sRNAs CcsR1-4 are co-transcribed with the *ccaF1* gene for an RNA-binding protein (group F). CcaF1 is involved in the RNase E-dependent processing of the *ccaF1*-*ccsr1-4* precursor RNA and also affects stability of other RNAs in *R. sphaeroides* [59]. Our data indicate different effects of RNase E and RNase III in the exponential and stationary phases. The underlying mechanisms (to date) are not known.

**Figure 9:**
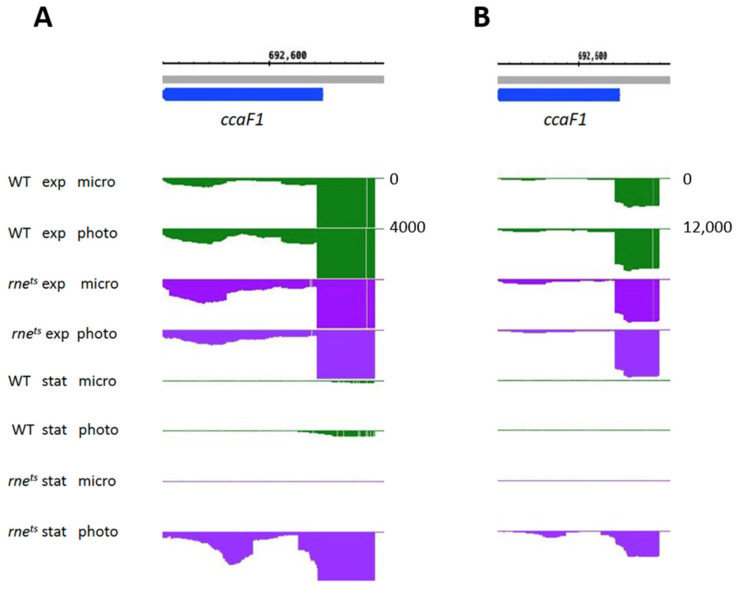
Normalized read coverage plot taken as a screenshot from the Integrated Genome Browser (IGB) showing reads for the *ccaF1* in the wild type and *rne^ts^* mutant. The *y*-axis count scale is indicated on the right side. A large scale is depicted in panel **B** (**A**: 0–4000 reads; **B**: 0–12,000 reads).

Finally, we also analyzed the expression of *hfq* and *ccaF1* (group F), two small RNA-binding proteins that affect photosynthesis gene expression in *R. sphaeroides* [12,26,59]. The important role of small proteins has become clear in recent years [62,73]. *hfq* expression did not respond to phototrophic growth conditions in the exponential and stationary phases and was not influenced by RNase E, RNase III, or StsR. In contrast, *ccaF1* expression was affected by growth conditions and was also affected by RNase E in the stationary phase. While *ccaf1* showed very low expression in the wild type in the stationary phase, much stronger expression was observed in the RNase E mutant in the stationary phase, albeit only under phototrophic conditions (Figure 9). This demonstrates the influence of RNase E on an RNA-binding protein that affects the levels of many RNAs in *R. sphaeroides* [59].

## 3. Materials and Methods

### 3.1. Growth of Bacterial Cultures

*R. sphaeroides* 2.4.1 [74] was cultivated in malate minimal medium [75] under microaerobic (25 mM dissolved oxygen, in the dark) or phototrophic conditions (no oxygen, illuminated with 60 W/m^2^ white light) at 32 °C.

To investigate the effects of RNase E, RNase III, and the sRNA StsR, three different mutant strains were used in this study. The thermosensitive RNase E mutant (*rne^ts^*) was generated via substitution of the native RNase E encoding gene (*rne*) with the *rne-3071* gene of *E. coli* [76], encoding a thermosensitive RNase E variant with reduced enzyme activity at 32 °C [17,27]. The *rnc***^−^** mutant was constructed via the exchange of two highly conserved amino acids within the catalytic center of RNase III and is characterized by the complete loss of RNase III cleavage activity [30]. For genomic deletion of StsR, the native *stsR* gene was replaced by a spectinomycin resistance cassette via homologous recombination [16].

To assess bacterial growth behavior, the optical density at 660 nm (OD660) of independent biological triplicates was measured photometrically using the Analytik Jena Specord 50 plus spectrophotometer (Analytik Jena, Jena, Germany).

### 3.2. Quantification of Photopigments and Photosynthetic Complexes

To quantify the amounts of photopigments produced per strain, 1 mL culture sample of independent biological triplicates was sedimented at 8000 rpm for 1 min. Subsequently, the cell pellets were resuspended in 50 µL ddH_2_O and thoroughly mixed with 500 µL methanol/acetone (1:1 *v*/*v*). Cell debris were removed via centrifugation at 13,000 rpm for 1 min. Absorbance of the supernatant was photometrically determined at λ = 770 nm (bacteriochlorphyll a) and λ = 484 nm (carotenoids), and pigment content was determined using the extinction coefficients 76 mM*^−^*^1^cm*^−^*^1^ (bacteriochlorophyll) and 128 mM*^−^*^1^cm*^−^*^1^ (carotenoids). Resulting values were normalized per cell density (OD_660_). Whole-cell absorbance spectra were recorded on a Specord 50 Plus spectrometer (Analytik Jena, Jena, Germany).

### 3.3. RNA Isolation and Quantificaiton

Total RNA was isolated using the hot phenol method [77], as described recently [28]. For northern blotting, 10 µg of total RNA (pooled from biological triplicates) was loaded per lane on denaturing 10% (*w*/*v*) polyacrylamide gels. Gel electrophoresis, blotting, hybridization with radiolabeled probes, and signal generation via phosphor imaging were performed as described earlier [30,59]. Radiolabeled deoxyoligonucleotides complementary to either StsR (sequence of oligonucleotide: 5′-GGACAGTGAAGGTAGAACGG-3′) or 5S rRNA (sequence of the oligonucleotide: 5′-CTTGAGACGCAGTACCATTG-3′) were used as probes for phosphor imaging.

For RNA-seq, residual DNA contaminations were degraded using the TURBO DNA-free Kit (Invitrogen/Thermo Fisher Scientific, Rockford, IL, USA) as described in the manufacturer’s manual. The sequencing libraries were constructed as described previously [16], using the NEBNext Multiplex Small RNA Library Prep Set for Illumina (NEB, Frankfurt am Main, Germany).

### 3.4. Bioinformatic Data Processing

Samples from biological triplicates of wild type, *rnc***^−^** mutant, *rne^ts^* mutant, and the ΔStsR strain, grown under aerobic, microaerobic, or phototrophic growth conditions, were analyzed via RNA sequencing. Curare v0.6 software [78] was used for the differential gene expression analysis. The raw sequencing data were adapter-trimmed and quality-filtered (phred score < 20 filtered out) with fastp v0.23.4 [79]. Afterward, the reads were aligned to the reference genome of *R. sphaeroides* (NC_007493.2, NC_007494.2, NC_009007.1, NC_007488.2, NC_007489.1, NC_007490.2, and NC_009008.1) using bowtie2 v2.5.2 [80] and saved as binary alignment maps (BAM) with Samtools v1.18 [81]. The alignments were summarized as bedgraph files using deeptools’ v3.5.4 bamCoverage function [82]. The coverage data (bedgraph files) were normalized by dividing each data point/base position by the total read number in each sample. For the screenshots, the replicates of each condition were averaged according to the arithmetic mean. The differential gene expression analysis used subreads featureCounts v2.0.6 [83] for counting and DESeq2 v1.40.2 [84] for the statistical analyses. Counting was conducted in featureCounts with “-s 1 -M --fraction” options on the “gene” level, and the NCBI GFF file for assembly GCF_000012905.2 was used as an annotation.

For further analysis, the identified genes were filtered using the GO (Gene Ontology) terms “0015979: photosynthesis”, “0015995: chlorophyll biosynthetic process”, “0016117: carotenoid biosynthetic process”, “0030076: light-harvesting complex”, “0030494: bacteriochlorophyll biosynthetic process”, “0036070: light-independent bacteriochlorophyll biosynthetic process”, “0033014: tetrapyrrole biosynthetic process”, “0006782: protoporphyrinogen IX biosynthetic process”, and “0006783: heme biosynthetic process” in the EMBL-EBI QuickGO database [31]. The filtering resulted in 89 unique genes, further referred to as “photosynthesis-related genes”. The following methods focus on the comparisons—“exponential versus stationary phase” and “microaerobic versus phototrophic condition”—of these selected genes.

The correlating DESeq2 results were filtered according to the following conditions: base mean > 10 and adjusted *p*-value < 0.05 for at least one comparison. All found photosynthesis-related genes fulfilled these conditions for both cases. To identify differentially expressed genes in both comparisons, log_2_ fold changes were visualized as boxplots using ggplot2 v3.5.0 [85] for all annotated genes in the corresponding DESeq2 comparisons and for the photosynthesis-related genes in the same DESeq2 comparisons. The selected photosynthesis-related genes were also visualized as log_2_ fold change heatmaps with pheatmap v1.0.12.

The log_2_ fold changes (from microaerobic to phototrophic) and their corresponding adjusted *p*-values (derived from the DESeq2 analyses), which served as input for Figure S3, Figure 4, and Figure 5 and Table 1 and Table 2, are listed in Supplementary Table S1 (for all annotated genes), Supplementary Table S2 (for photosynthesis-related and further selected genes), and Supplementary Table S3 (gene expression changes between wild type and mutant strains).

## 4. Conclusions

**Figure 10:**
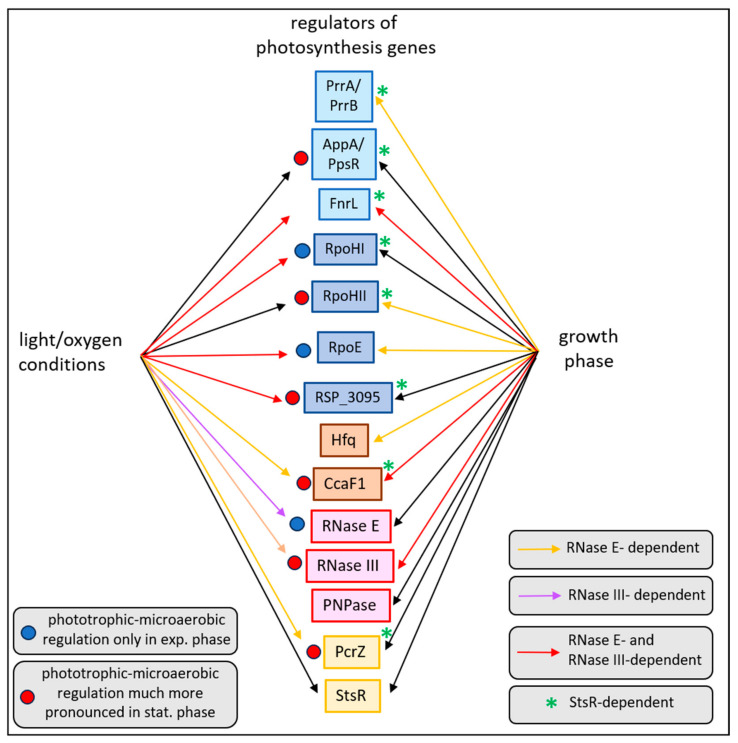
Overview on the effects of growth conditions (from microaerobic to phototrophic) and growth phases (from exponential to stationary) on the expression of genes important for regulation of photosynthesis genes. Arrows indicate effects dependent on either light/oxygen conditions or the growth phase. Orange, purple, and red color indicates an additional dependency on RNase E, RNase III, or both RNase E and RNase III, respectively.

Although the ability to perform photosynthesis offers an alternative pathway for ATP production when oxygen levels are limited, the production of pigments can be harmful in the presence of oxygen. Consequently, the expression of photosynthesis genes is controlled by oxygen and light conditions, and many of the underlying molecular mechanisms have been elucidated in the past. The present study reveals that growth phases have a strong impact on adaptation to changing environmental conditions. As outlined in Figure 10, many regulators of photosynthesis genes show differential expression in exponential and stationary phases. The sRNA StsR affects the growth-phase-dependent expression of all the regulators show and, consequently, the expression of photosynthesis genes.

In addition, this study demonstrates the important role of RNases not only in adaptation to growth conditions but also in adaptation to growth phases. All colored arrows in Figure 10 indicate the influence of at least one RNase in regulation. All regulators shown are also influenced by StsR, underlining the central role of this sRNA in the adaptation of *R. sphaeroides* to environmental conditions. Studies on different bacterial systems in recent years have revealed the important role of sRNAs as key mediators in large regulatory networks in bacteria (e.g., reviewed in [86,87]).

## Figures and Tables

**Table 1:** Mean log_2_ fold change in expression within a gene cluster (as outlined in the main text) based on DESeq2 analysis of all annotated genes between phototrophic and microaerobic growth conditions. The number of genes within a cluster is given in row 2. Strong changes in average expression (log_2_ fold change > 1.6 or <−1.6) are marked in red (strong positive change) and blue (strong negative change). A summary of all corresponding log_2_ fold changes and their adjusted *p*-values obtained from the DESeq2 analyses is provided in Supplementary Table S1 (for all analyzed genes) and Supplementary Table S2 (for selected genes).

	Gene Expression Change from Microaerobic to Phototrophic Given as log_2_ Fold Change							
	Wild Type		*rne^ts^*		*rnc* ^−^		ΔStsR	
	Exp.	Stat.	Exp.	Stat.	Exp.	Stat.	Exp.	Stat.
PS gene cluster (*n* = 38)	0.7	2.0	0.5	2.7	0.5	2.2	0.5	1.6
motility (*n* = 63)	−1.7	−0.7	2.8	−2.2	−0.5	−0.4	0.3	−1.1
*dcw* gene cluster (*n* = 24)	−0.2	0.5	0.1	0.3	−0.3	0.8	−0.3	0.6
ribosomal gene cluster (*n* = 37)	−0.4	1.3	0	0.9	−0.4	1.1	0	0.7
*nuo* gene cluster (*n* = 22)	−0.5	0.1	−0.5	−0.7	−0.5	−0.2	0.7	0.1

**Table 2:** Expression change (based on DESeq2) of selected genes with a role in the regulated formation of photosynthetic complexes. RNAs with log_2_ fold change ≥ 1 are highlighted in red; RNAs with log_2_ fold change ≤ −1 are highlighted in blue. When the analyzed RNA comprises more than one gene, the mean is given. We considered expression as dependent on RNase E, RNase III, or StsR when the calculated fold change in expression differed by at least a factor of 2 compared to the wild type changes (marked in bold). *: adjusted *p*-value < 0.05; n.a.: this RNA is missing or altered in the mutant. A summary of all corresponding log_2_ fold changes and their adjusted *p*-values obtained from the DESeq2 analyses is provided in Supplementary Table S1 (for all analyzed genes) and Supplementary Table S2 (for selected genes).

		Gene Expression Change from Microaerobic to Phototrophic Given as log_2_ fold Change							
		Exponential Phase				Stationary Phase			
		WT	*rne^ts^*	*rnc* ^−^	ΔStsR	WT	*rne^ts^*	*rnc* ^−^	ΔStsR
***A***	*pucBAC*	1.5	2.2 *	1.4	1.1	2.8 *	4.5 *	4.4 *	3.4 *
	*pufQ-X*	1.6 *	0.8 *	1.0	1.2 *	0.8	3.5 *	**2.0**	1.6 *
	*hemZ*	−0.4	0.1	−0.7 *	0.0	3.7 *	3.7 *	3.4 *	2.9 *
	*hemA*	1.4 *	**0.3**	0.9 *	0.6 *	3.3 *	3.2 *	3.0 *	2.1 *
	*crtE*	1.2 *	**−0.2**	0.7 *	0.7 *	4.8 *	3.0 *	4.6 *	3.5 *
	*crtIB*	1.1 *	**−0.4**	0.8 *	0.5	1.8 *	3.4 *	1.8 *	1.1 *
	*crtD*	1.5 *	0.6 *	1.0 *	0.6 *	3.0 *	2.4 *	2.7 *	2.4 *
	*bchCXYZ*	0.7	0.5	0.3	0.4	2.9 *	4.5 *	3.1 *	2.3 *
	*bchEJ*	0.6	**−0.4**	0.4	0.9 *	4.6 *	2.6 *	4.8 *	3.7 *
	*crtA-bchI*	1.3 *	1.1 *	0.9 *	0.9 *	2.3 *	3.3 *	2.4 *	1.7 *
	*RSP_0290*	1.1 *	0.9 *	0.9 *	0.8 *	2.3 *	3.3 *	2.9 *	1.8 *
	*fbcFBC*	0.8	0.6 *	0.5	0.6	1.8 *	4.1 *	2.0 *	2.3 *
***B***	*prrA*	0.4	−0.1	−0.1	0.0	0.3	1.4 *	0.8 *	0.2
	*prrB*	0.2	0.1	0.1	−0.3 *	0.3	−0.5 *	0.3	1.0 *
	*fnrL*	−1.0 *	−0.3	−0.4 *	−0.6 *	−1.1 *	−0.3	−0.7 *	**−0.1**
	*appA*	−0.2	−0.6 *	−0.7 *	−0.1	3.4 *	2.4 *	2.8 *	1.9 *
	*ppsR*	−0.9 *	−0.3	−0.4 *	−0.7 *	0.1	0.6 *	0.5 *	0.0
***C***	*rne*	−1.0 *	n.a.	−0.5 *	−1.0 *	0.4 *	n.a.	0.7 *	0.7 *
	*rnc*	−0.7 *	0.2	n.a.	−0.4 *	1.8 *	**0.0**	n.a.	1.0 *
	*pnp*	0.1	0.8 *	0.1	0.4 *	−0.5 *	0.3	0.0	−0.8 *
***D***	*rpoHI*	2.2 *	1.2 *	1.5 *	1.1 *	0.1	−2.1 *	−0.8 *	0.1
	*rpoHII*	0.3	−0.6 *	0.2	**1.3** *	0.7 *	0.8 *	0.6 *	**2.9**
	*rpoE1*	0.7 *	0.2	0.5 *	0.7 *	2.1 *	0.7 *	1.7 *	2.5 *
	*RSP_3095*	0.1	−0.2	−0.1	−0.2	2.3 *	0.6 *	**0.3**	**−0.4**
***E***	*pcrZ*	0.0	0.4 *	0.4	2.6 *	0.8 *	−1.7 *	0.0	−0.4 *
	*stsR*	0.6 *	0.9 *	0.0	n.a.	1.4 *	1.4	1.7 *	n.a.
	*upsM*	−1.1 *	−1.0 *	−0.5	**−0.3**	−1.6 *	**−0.6**	**−0.2**	**0.3**
	*ccsR1*	1.7 *	0.7 *	**0.5**	1.6 *	3.1 *	5.2 *	2.2 *	5.2 *
***F***	*hfq*	0.2	0.2	0.2	0.2	-0.1	0.4 *	0.3 *	0.4 *
	*ccaF1*	0.7 *	0.7	**−0.3**	−0.1	2.0 *	6.3 *	2.9 *	4.2 *

## Data Availability

The RNA-seq data are available in the NCBI gene expression omnibus (GEO) repository. The data for wild type and RNase E mutant grown to exponential phase are listed in GSE200990 (https://www.ncbi.nlm.nih.gov/geo/query/acc.cgi?acc=GSE200990 (accessed on 15 July 2024). Data of the RNase III mutant from exponential phase are listed in GSE236804 (https://www.ncbi.nlm.nih.gov/geo/query/acc.cgi?acc=GSE236804 (accessed on 15 July 2024)). The RNA sequencing of the ΔStsR strain and the stationary phase data of the other strains are listed in GSE271933 (https://www.ncbi.nlm.nih.gov/geo/query/acc.cgi?acc=GSE271933 (accessed on 15 July 2024)).

## Supplementary Materials

The following supporting information can be downloaded at https://www.mdpi.com/article/10.3390/ijms25169123/s1.
