# Supplementary material for: The Impact of the Major Endoribonucleases RNase E and RNase III and of the sRNA StsR on Photosynthesis Gene Expression in Rhodobacter sphaeroides Is Growth-Phase-Dependent
Source: Int J Mol Sci. 2024 Aug 22;25(16):9123. doi: 10.3390/ijms25169123 (PMC11354728; doi:10.3390/ijms25169123)
Supplement: Supplementary file 1 [file ijms-25-09123-s001.zip › Boerner2024_Supplement.pdf]

Supplementary Material

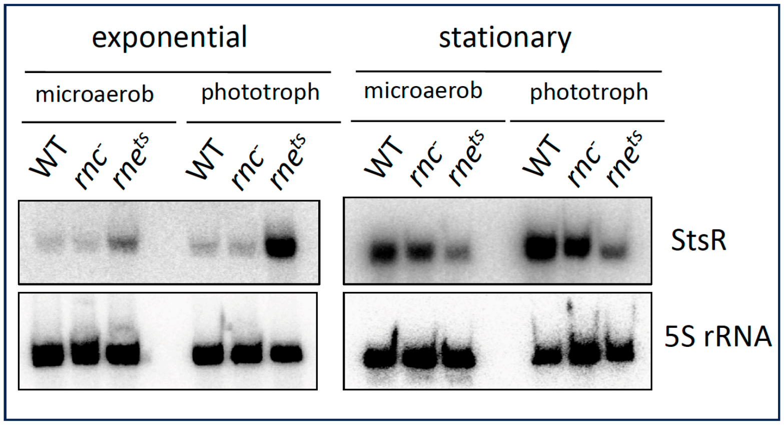

Figure S1. Northern blot showing the steady state level of the sRNA StsR in wild type (WT), the *rne*[ts] and the *rnc*- strain under different growth conditions (microaerobic and phototrophic) and in different growth phases (exponential and stationary). 5S rRNA serves as a loading control.

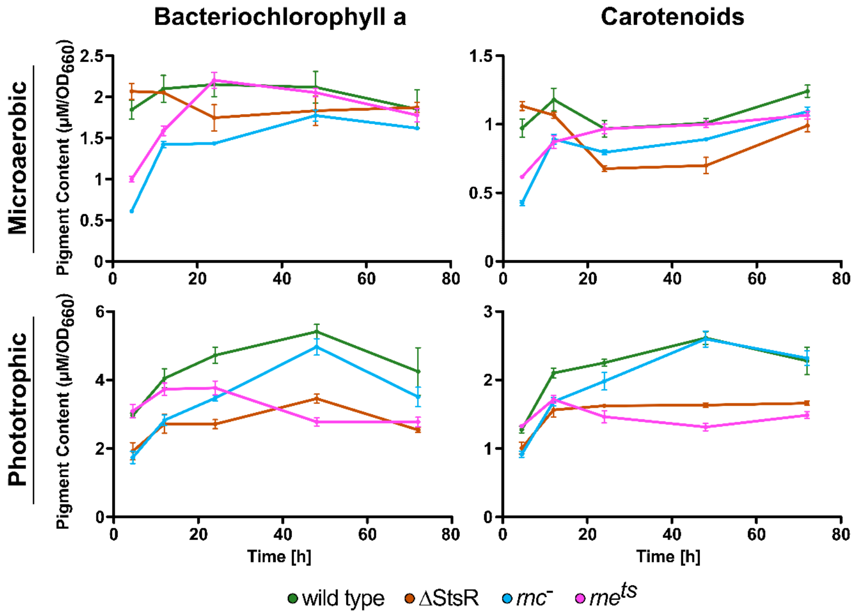

Figure S2. Quantification of photopigments at various time points from exponential to late stationary growth phase. Bacterial strains were cultivated under microaerobic (top panels) or phototrophic (bottom panels) growth conditions. Samples for methanol/acetone extraction of photopigments were harvested after 4.5 h, 12 h, 24 h, 48 h or 72 h of growth. The mean contents of bacteriochlorophyll a (left panels) and carotenoids (right panels) of independent biological triplicates, cell count normalized to OD_{660}, are plotted. The standard deviation of the mean is indicated as error bar.

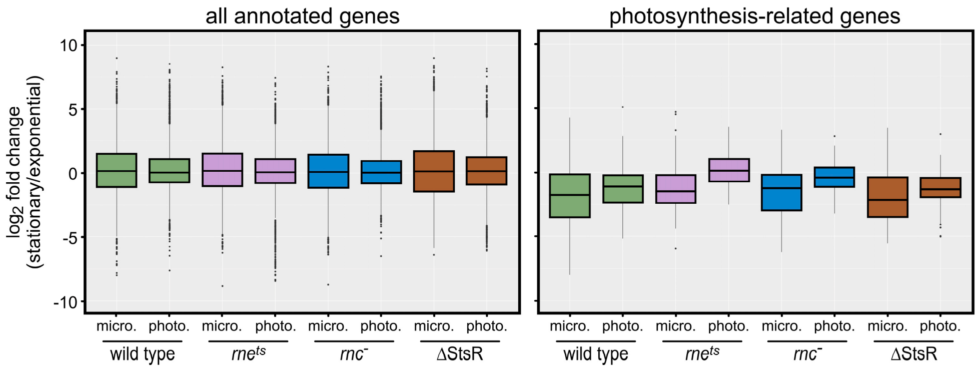

Figure S3. Box plots showing the distribution of the $\log_2$ fold changes of all annotated genes (left panel) and of photosynthesis-related genes as outlined in the main text (right panel) from exponential to stationary phase. The $\log_2$ fold changes are taken from the corresponding DESeq2 analyses as described in the materials and methods section.
